# A Preliminary Integrated Metabolomics Analysis Identifies Retrograde Endocannabinoid Signaling Shift in Severe OSA Patients

**DOI:** 10.1007/s11325-025-03425-w

**Published:** 2025-07-31

**Authors:** Li Han, Jinjin Liu, Juan Xu, Xiaoyan Li, Rui Chen

**Affiliations:** 1 PCCM, The Second Affiliated Hospital of Soochow University, Suzhou, 215000 China; 2 PCCM, Shanghai University of Medicine & Health Science Affiliated Zhoupu Hospital, Shanghai, 201318 China; 3 Department of Respiratory Medicine, Putuo Hospital, Shanghai University of Traditional Chinese Medicine, Shanghai, 200062 China; 4 Department of Respiratory Medicine, The First People’s Hospital of Yancheng City, The Yancheng Clinical College of Xuzhou Medical University, Yancheng, 224005 China

**Keywords:** Obstructive sleep apnea, Reactive oxygen species, Metabolomics, Sleep fragmentation, Retrograde endocannabinoid signaling

## Abstract

**Purpose:**

The systemic influence of obstructive sleep apnea(OSA), particularly in those with severe OSA (sOSA), has frequently been disregarded. An investigation was conducted to examine the variations of reactive oxygen species (ROS) and metabolomic drifts, as well as their interrelations, in adult OSA patients.

**Methods:**

A total of 22 male patients were enrolled after undergoing polysomnography (PSG), and blood samples were obtained. Following the measurement of ROS levels in polymorphonuclear neutrophils (PMNs), plasma samples were combined and analyzed using both Gas Chromatography-Mass Spectrometry (GC–MS) and Liquid Chromatography-Mass Spectrometry (LC–MS).

**Results:**

Patients with sOSA had a higher body mass index (BMI) (p = 0.005) and more negative PSG measures compared to those with mild/moderate OSA (mOSA). Although there was no remarkable difference in neutrophil ROS between the mOSA and sOSA group, further analysis indicated sleep fragmentation was associated with ROS production(p = 0.043 for N3%, p < 0.01 for Arousal Index). Integrated metabolomics analysis revealed significant alterations in 59 metabolites from GC–MS and 27 metabolites from LC–MS in sOSA patients. These substantial changes perturbed various pathways, including the FoxO signaling pathway and its upstream PI3K-Akt signaling pathway, and so on. Notably, the retrograde endocannabinoid signaling pathway was the sole common pathway identified in both GC–MS and LC–MS data.

**Conclusions:**

This study sheds light on the systemic impact of OSA, particularly in severe cases, by uncovering extensive disturbance of metabolic pathways. The identification of retrograde endocannabinoid signaling shifts in severe OSA adults highlights its potential impact in the sleep disorder and its broader repercussions in the body.

## Introduction

Obstructive sleep apnea (OSA) is a common global disorder that is often neglected. The estimated incidence of OSA in American people aged 30–69 is quite significant, reaching up to 33.2% (apnea-hyponea index, AHI ≥ 5) and 14.5% (AHI ≥ 15). This prevalence varies by gender, with 33.9% in men and 17.4% in women [1]. Similarly, epidemiological research conducted in China showed a high OSA prevalence of 23.6% (AHI ≥ 5) and 8.8% (AHI ≥ 15) among adults, impacting a large section of the population [1]. OSA is distinguished by intermittent hypoxia (IH) and sleep fragmentation, both of which are prominent pathophysiologic characteristics. Excess reactive oxygen species (ROS) would be produced in response to IH-induced oxidative stress (OS), resulting in systemic health consequences. OSA has been associated with a variety of comorbidities, including cardiovascular disease (hypertension, ischaemic heart disease, and stroke), metabolic disorders (metabolic syndrome or diabetes mellitus), depression, cognitive impairment, and even an increased risk of traffic accidents [2–4]. Comorbidities are more common in severe OSA patients, necessitating further research into the systemic influence on the milieu interne, notably in terms of metabolic abnormalities [3, 5].

Metabolomics, using high-resolution and high-sensitivity spectrometry, provides a comprehensive view of endogenous tiny metabolites (≤ 1000 Da) in different physiological states or phenotypes. These metabolites, which influence the functional phenotypes of animals or cells, can be studied with liquid chromatography-mass spectrometry (LC–MS) and gas chromatography-mass spectrometry (GC–MS) [6]. Previous investigations employing metabolomic methods found metabolic anomalies in the peripheral blood of OSA patients. Several different metabolites, including fatty acids and phospholipids, various amino acids, deoxy sugars, arachidonic acids, and other micromolecular intermediate metabolites, were shown to be drastically altered [7–11].

Most of the research mentioned above were meant to compare OSA adults to healthy controls, but fewer paid attention to metabolomic variations among OSA patients of varying severity. Clinically, severe OSA is more likely to result in complications and a poor prognosis [12]. The distinctive aspects of severe OSA, including ROS-related effects on different phenotypes, remain poorly understood. This study aims to discover metabolomic alterations, particularly in severe OSA patients, and investigate the links between metabolites, ROS levels, and clinical indicators.

## Material and Methods

### Recruitment and sample collection

**Fig. 1:**
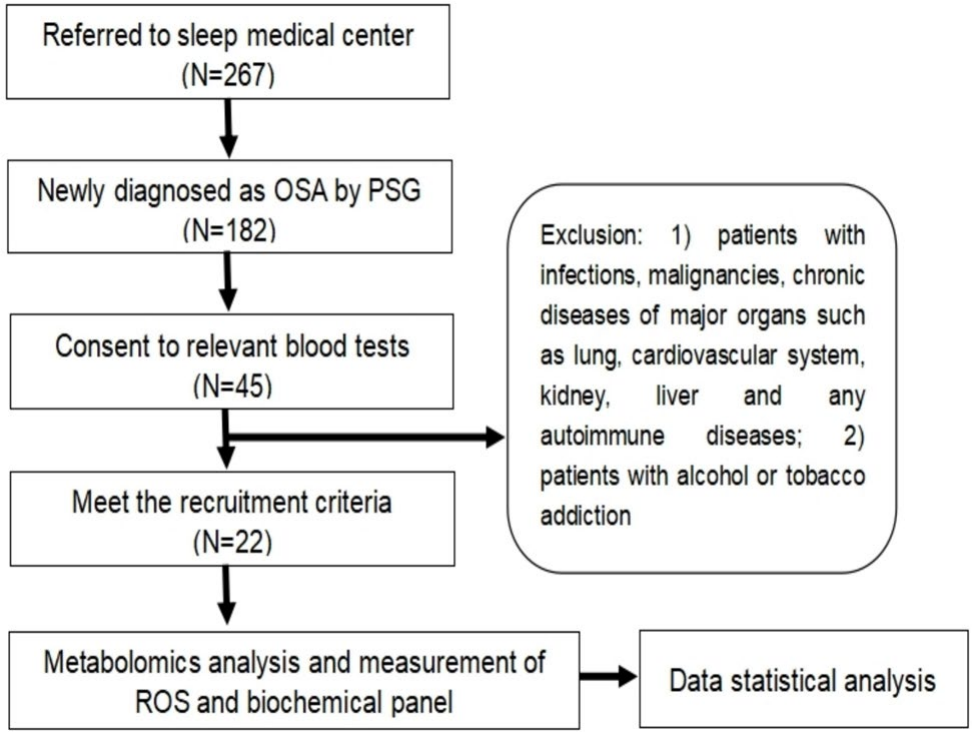
Flow Chart of the Study Recruitment

22 male patients were recruited for polysomnography (PSG) at the Sleep Medicine Center of Soochow University's Second Affiliated Hospital. The patients were all of Chinese Han ethnicity. Patients aged 18 to 65 yr with an OSA diagnosis based on the 2016 American Academy of Sleep Medicine (AASM) revised criteria (AHI ≥ 5) who were fasting in the morning were eligible. Patients with infections, cancer, chronic disorders of major organs, hypertension, diabetes, autoimmune diseases, or a history of smoking or alcohol usage(20 g/day for men and 10 g/day for women) were excluded from the study (Fig.1). Of them, 20 were newly diagnosed and had not received any treatment, while two had been previously diagnosed but had not received treatment within the previous year. The 22 patients were divided into two groups based on their AHI: mild or moderate OSA (mOSA) defined as AHI < 30, and severe OSA (sOSA) defined as AHI ≥ 30 (according to AASM recommendations). Fasting blood samples were obtained in the morning, processed, and stored at −70 °C. The study was authorized by the Research Ethics Committee of Soochow University's Second Affiliated Hospital in Suzhou, China (JD-LK-2018–004-02), and all patients provided informed consent.

### ROS and biological detection

Anticoagulant peripheral blood (2.7 ml) was processed to isolate polymorphonuclear neutrophils (PMNs) following the instructions of the kit (P9040, Beijing Solarbio Science & Technology Co.,Ltd., China), with additional procedures carried out according to the kit's instructions (S0033, Beyotime Biotech Inc, Shanghai, China). The relevant biochemical indices in serum were determined using the iCARE-2000 platform (Sinocare, Changsha, China).

### LC–MS-based untargeted metabolomics analyses

Metabolomics analysis comprised adding 10μL of mixed internal standards to 150μL of thawed plasma samples, followed by extraction with ice-cold methanol and acetonitrile (2:1 v/v). The samples were vortexed for 1 min, sonicated for 10 min, then incubated at −20 °C for 5 h. After centrifugation at 13,000 rpm and 4 °C for 10 min, the supernatants (150 μL) were collected and filtered through 0.22 μm microfilters before being transferred to LC vials. The ACQUITY UPLC I-Class system (Waters Corporation, Milford, USA) and the VION IMS QTOF Mass Spectrometer (Waters Corporation, Milford, USA) were utilized for analysis in both ESI positive and negative modes. Chromatographic separation was carried out using an ACQUITY UPLC BEH C18 column and a gradient elution system. Data acquisition parameters were tuned to ensure correct analysis, and quality control samples were used to check data reproducibility.

### GC–MS-based untargeted metabolomics analyses

After adding 20μL of internal standards to 150μL of thawed plasm sample, extraction was performed with 450μL of ice-cold methanol and acetonitrile (2:1, v/v). The samples were then vortexed, sonicated, incubated, and centrifuged. The supernatants were derivatized and processed via a DB-5MS capillary column with helium as the carrier gas. The temperature program was tuned to provide efficient separation and detection.

### Data preparation and statistical analysis

Aligned with the Human Metabolome Database (HMDB, http://www.hmdb.ca/) and Shanghai Lu-Ming Biotech Co's self-built database. Raw data from Ltd. (Shanghai, China) were processed with Progenesis QI software (Waters Corporation, Milford, USA). The differential metabolite (DM) data were then screened and processed using principal component analysis (PCA), partial least squares discriminant analysis (PLS-DA), orthogonal partial least squares discriminant analysis (OPLS-DA), and t-test. Metabolites with Variable importance in projection (VIP) > 1.0 and P < 0.05 were classified as DMs (false discovery rate,i.e.FDR < 0.1). The baseline data was evaluated using a t-test, and findings were provided as mean ± SD. P < 0.05 was regarded as statistically significant.

## Results

### Clinical and PSG characteristics with ROS measurement

The average age was 37.73 ± 9.11 yr, with a mean BMI of 27.68 ± 3.76 kg/m^2^. The average AHI was 16.95 ± 9.98 for mOSA and 62.92 ± 24.13 for sOSA (p < 0.001). The mOSA and sOSA groups showed significant differences in BMI (p = 0.005), ODI (p < 0.001), T90%(p = 0.008), LO2(p = 0.001), N1% (p = 0.018), N3% (p = 0.003), RERA(p = 0.001), and arousal index (p < 0.001) (refer to Table 1). The nonSWS%, i.e. (N1 + N2)/TST(total sleep time), showed a difference between the two groups (p = 0.015). Most biochemical measures did not reveal a significant change, except for HDL-C (p = 0.012). Patients with sOSA had higher BMI and worse PSG values than mOSA patients. It was shown that the sOSA group had not only heavier hypoxemia performance, but also more fragmented sleep with more frequent arousals (Table 1). Meanwhile, there was no significant difference in neutrophil ROS levels between the mOSA and sOSA groups, as well as a few common biochemical indices such as fasting plasma glucose (FPG), homocysteine (HCY), total cholesterol (Tcho), triglycerides (TG), and low-density lipoprotein cholesterol (LDL-C).

**Table 1:** PSG and clinical Characteristics of the Participants

	All(n = 22)	mOSA(n = 10)	sOSA(n = 12)	p-value
Age(year)	37.73 ± 9.11	36.20 ± 7.44	39.00 ± 10.45	0.486
BMI(kg/m^2^)	27.68 ± 3.76	25.40 ± 1.30	29.58 ± 4.12	0.005*
SBP(mmHg)	123.82 ± 14.82	122.90 ± 17.34	124.58 ± 13.12	0.798
DBP(mmHg)	83.41 ± 9.13	80.90 ± 8.09	85.50 ± 9.75	0.249
mBP(mmHg)	96.89 ± 10.25	94.91 ± 10.58	98.53 ± 10.12	0.422
AHI(/h)	42.02 ± 29.94	16.95 ± 9.98	62.92 ± 24.13	< 0.001*
ODI(/h)	33.35 ± 27.28	12.80 ± 10.57	50.47 ± 25.02	< 0.001*
T90%	14.71 ± 19.85	3.05 ± 2.91	24.42 ± 22.78	0.008*
LO2(%)	72.86 ± 13.27	82.10 ± 5.57	65.17 ± 12.98	0.001*
SL(min)	8.91 ± 6.35	9.90 ± 7.34	8.08 ± 5.58	0.517
N1%	19.73 ± 14.39	12.07 ± 11.05	26.12 ± 14.05	0.018*
N2%	47.77 ± 10.57	49.23 ± 9.21	46.56 ± 11.86	0.568
N3%	12.38 ± 9.35	18.41 ± 8.01	7.36 ± 7.32	0.003*
REM%	20.12 ± 7.30	20.33 ± 6.05	19.94 ± 8.47	0.905
nonSWS%	67.51 ± 11.33	61.30 ± 7.89	72.68 ± 11.40	0.015*
REML(min)	134.16 ± 101.76	107.80 ± 66.60	156.13 ± 122.40	0.278
RERA(/h)	23.30 ± 24.71	5.20 ± 4.83	38.38 ± 24.51	0.001*
SE(%)	78.31 ± 15.52	80.92 ± 14.58	76.13 ± 16.56	0.484
ArI(/h)	34.98 ± 25.75	16.24 ± 10.89	50.59 ± 24.16	< 0.001*
WASO(min)	91.07 ± 76.26	70.00 ± 62.67	108.63 ± 84.56	0.246
ESS	9.18 ± 6.19	7.50 ± 5.26	10.58 ± 6.78	0.254
FPG(mmol/L)	4.82 ± 0.76	4.77 ± 0.93	4.86 ± 0.63	0.941
HCY(umol/L)	11.91 ± 4.91	11.08 ± 4.38	12.61 ± 5.39	0.520
UA(umol/L)	406.47 ± 60.93	397.14 ± 72.61	414.24 ± 51.28	0.240
LDL-C(mmol/L)	3.66 ± 0.71	3.54 ± 0.66	3.76 ± 0.76	0.266
Tcho(mmol/L)	4.68 ± 0.84	4.47 ± 0.74	4.86 ± 0.90	0.197
TG(mmol/L)	1.70 ± 1.28	1.39 ± 0.67	1.96 ± 1.61	0.286
HDL-C(mmol/L)	0.93 ± 0.25	1.01 ± 0.18	0.86 ± 0.29	0.012*
ROS	0.57 ± 0.12	0.55 ± 0.13	0.60 ± 0.11	0.346

Abbreviations: SBP,systolic blood pressure; DBP,diastolic blood pressure; mBP,mean blood pressure; T90%, the percentage of sleep time spent with SpO_2_ < 90%; LO2(%), minimal saturation; SL,sleep latency; N1%, the percentage of stage N1; N2%, the percentage of stage N2; N3%, the percentage of stage N3; REM%, the percentage of stage REM; nonSWS%, the percentage of stage N1and N2; REML, REM latency; RERA, respiratory effort related arousal(/hour); SE(%),sleep effectiveness; ArI, arousal index; SL,sleep onset latency; WASO, wake after sleep onset; ESS, Epworth sleepiness scale; FPG, fast plasma glucose; HCY, homocysteine; UA, uric acid; LDL-C, low density lipoprotein cholesterol; Tcho, total cholesterol; TG, triglyceride; HDL-C, high density lipoprotein cholesterol

### Identification of DMs in plasma using GC–MS and LC–MS

**Fig. 2:**
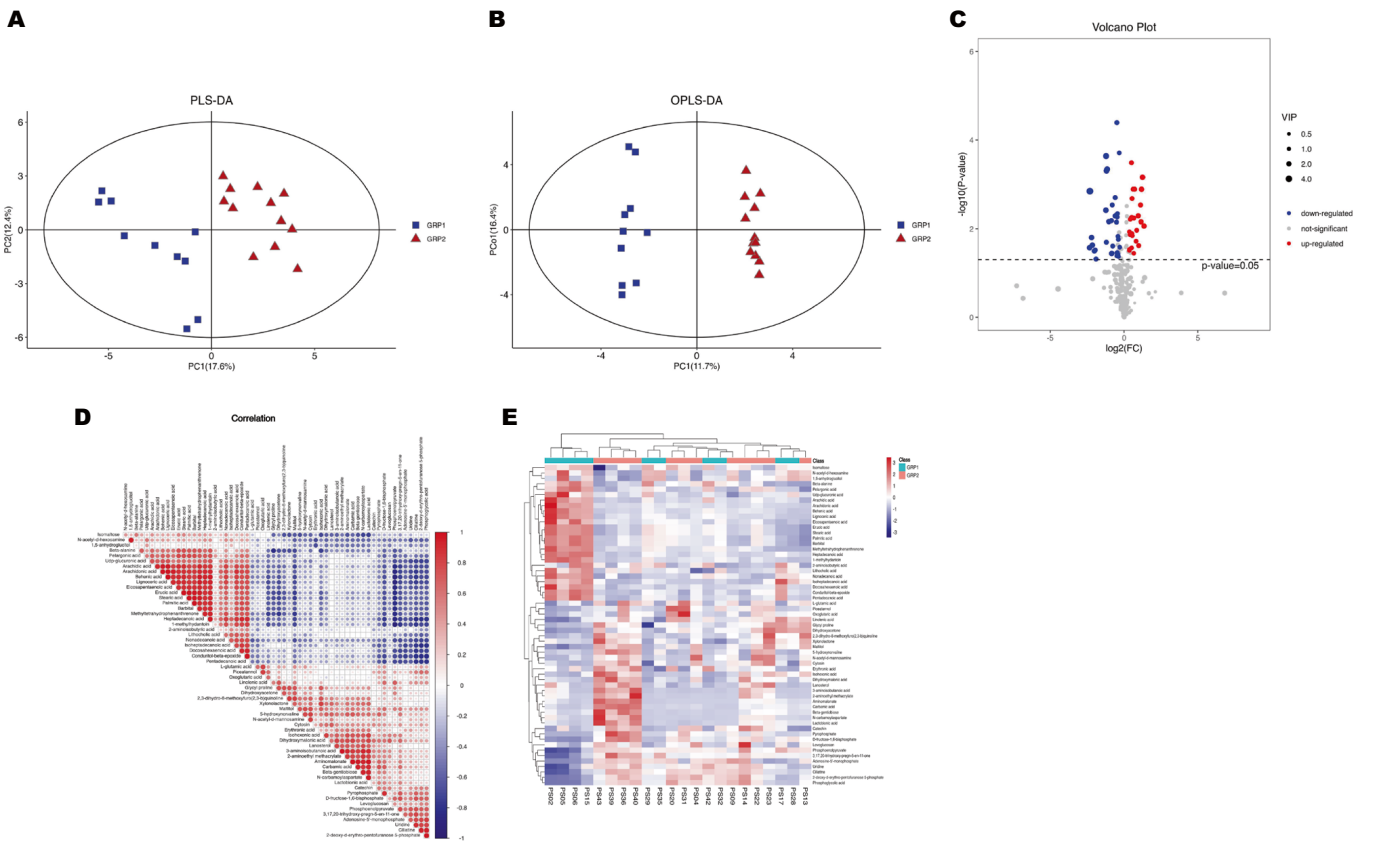
Visualization Plots of Screening Differential Metabolites by GC–MS. Scatter plots for PLS-DA (R^2^X = 0.384, R^2^Y = 0.973, Q^2^ = 0.716) and OPLS- DA (Q^2^ = 0.719, R2 = 0.96) models built from untargeted GC-MS analysis, showing separation between mOSA and sOSA patients in (**A**) and (**B**). Volcano plots of the FC (defined as FC = 1.25) and p value (defined as 0.05) for DMs between the two groups shown in (**C**). Red dots represent significantly upregulated (FC > 1.25) molecules and blue dots represent significantly downregulated (FC < 0.8) molecules. Correlations between the DMs demonstrated in (**D**). The color scale illustrates the degree of correlation and ranges from red to blue, indicating positive and negative correlations respectively. The important DMs are displayed in the heatmap of (**E**), with the red color indicating an increased trend and the blue color indicating a decreased trend compared to mOSA(GRP1)

The PLS-DA GC–MS model clearly discriminated sOSA patients from mOSA patients, showing that the two groups had different metabolites (DM) (Fig. 2A). OPLS-DA models (VIP > 1.0 and p < 0.05) revealed distinct differences between mOSA and sOSA patients (Fig. 2B). Based on the combination of OPLS-DA and t-test, 59 DMs were discovered using GC–MS (Fig. 2C). The volcano plot and heatmap showed metabolic differences between the two phenotypes (Fig. 2C, 2E). Table 2 shows the top 16 DMs with VIP > 1.8 and p < 0.05. The relationships between DMs were shown in Fig. 2D. The DMs with mutually strong links, such as arachidic acid, arachidonic acid, behenic acid, lignoceric acid, eicosapentaenoic acid, erucic acid, stearic acid, palmitic acid, methyltetrahydrophenanthrenone, and heptadecanoic acid, mainly belonged to close-knit metabolic pathways such as long-chain fatty acid catabolism and anabolism, as well as arachidonic acid metabolism.

**Table 2:** the top DMs between groups in GC–MC

	Retension Time	m/z	Compounds	VIP	p-value	FC	V
1	16.51	451.10	Pyrophosphate	4.25	1.43E-3	0.20	**↓**
2	27.34	357.15	2-deoxy-d-erythro-pentofuranose 5-phosphate	2.96	2.30E-4	0.43	**↓**
3	27.34	357.14	Phosphoglycolic acid	2.81	4.59E-4	0.45	**↓**
4	20.94	299.08	D-fructose-1,6-bisphosphate	2.57	4.96E-4	0.44	**↓**
5	22.11	117.03	Heptadecanoic acid	2.34	6.95E-4	2.38	**↑**
6	21.82	327.25	Isoheptadecanoic acid	2.23	1.29E-3	2.27	**↑**
7	25.13	397.38	Behenic acid	2.01	8.69E-3	2.55	**↑**
8	21.91	174.11	3-aminoisobutanoic acid	2.19	1.57E-2	0.22	↓
9	13.69	147.05	Aminomalonate	2.14	2.34E-2	0.23	↓
10	22.46	174.12	2-aminoethyl methacrylate	2.10	2.68E-2	0.20	↓
11	28.70	243.10	Adenosine-5'-monophosphate	2.23	3.61E-2	0.56	↓
12	26.14	117.03	Lignoceric acid	1.91	7.00E-3	2.22	**↑**
13	12.61	116.08	N-carbamoylaspartate	1.93	3.16E-2	0.25	↓
14	17.64	217.11	Udp-glucuronic acid	1.90	6.16E-3	1.95	**↑**
15	7.24	200.09	2,3-dihydro-8-methoxyfuro(2,3-b)quinoline	1.88	6.93E-3	0.49	**↓**
16	13.13	197.02	Methyltetrahydrophenanthrenone	1.82	2.94E-3	2.17	**↑**

**Fig. 3:**
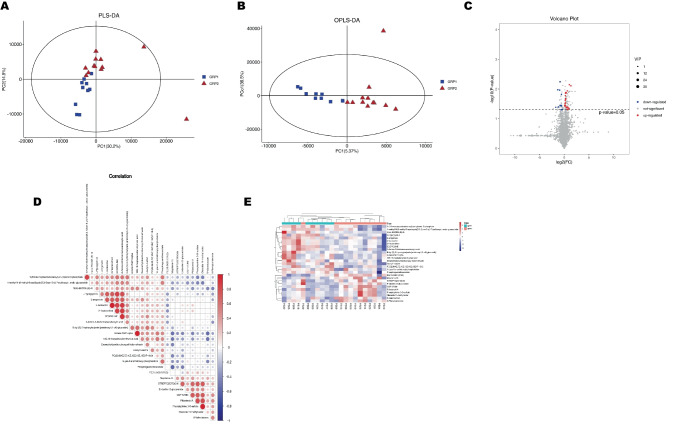
Visualization Plots of Screening Differential Metabolites by LC–MS. Scatter plots for PLS-DA (R^2^X = 0.597, R^2^Y = 0.765, Q^2^ = −0.002) and OPLS- DA (Q^2^ = −0.625, R^2^ = 0.771) models built from untargeted LC–MS analysis, showing a degree of separation between mOSA and sOSA patients in (**A**) and (**B**). Volcano plots of the FC (defined as FC = 1.25) and p value (defined as 0.05) for DMs between the two groups shown in (**C**). Red dots represent significantly upregulated (FC > 1.25) molecules and blue dots represent significantly downregulated (FC < 0.8) molecules. Correlations between the DMs demonstrated in (**D**). The color scale illustrates the degree of correlation and ranges from red to blue, indicating positive and negative correlations respectively. The important DMs are displayed in the heatmap of (**E**), with the red color indicating an increased trend and the blue color indicating a decreased trend compared to mOSA(GRP1)

In the PLS-DA model of LC–MS, sOSA patients also differed from mOSA patients, indicating DMs between the two groups (Fig. 3A). The OPLS-DA model (VIP > 1 and p < 0.05) demonstrated similar differentiation (Fig. 3B). LC–MS found 27 DMs (Fig. 3C, 3E). The top 13 DMs were identified with VIP > 1.2 and p < 0.05 (refer to Table 3). The correlations among pertinent DMs were indicated in Fig. 3D.

**Table 3:** the top DMs between groups in LC-MC

	Retension Time	m/z	Compounds	VIP	p-value	FC	V
1	1.28	132.10	L-Isoleucine	9.377	3.69E-2	1.12	**↑**
2	12.67	828.55	PC(22:6(4Z,7Z,10Z,13Z,16Z,19Z)/P-16:0)	9.34	1.35E-2	1.26	**↑**
3	10.81	776.54	PE(15:0/20:1(11Z))	5.05	3.67E-2	0.66	**↓**
4	4.02	453.25	1-(18-mercaptooctadecanoyl)-sn-glycerol 3-phosphate	4.17	4.86E-2	1.57	**↑**
5	12.48	457.24	1-Lyso-2-arachidonoyl-phosphatidate	3.52	4.15E-2	1.38	**↑**
6	2.18	100.08	δ-Valerolactam	2.57	5.00E-2	0.65	**↓**
7	1.28	133.10	2-Heptanethiol	2.48	3.63E-2	1.12	**↑**
8	3.55	227.08	L-arogenate	1.84	3.13E-2	1.13	**↑**
9	6.59	292.05	Phenylephrine 3-O-sulfate	1.58	4.04E-2	0.43	**↓**
10	7.42	550.25	Endoxifen O-glucuronide	1.55	1.06E-2	0.41	**↓**
11	6.83	369.23	6-Keto-PGF1α	1.40	7.80E-3	2.58	**↑**
12	4.64	472.24	Butyl (S)−3-hydroxybutyrate [arabinosyl-(1- > 6)-glucoside]	1.32	4.19E-2	1.68	**↑**
13	4.31	245.19	Leucyl-Leucine	1.24	1.16E-2	1.53	**↑**

### Pathway enrichment analysis of DMs using GC–MS and LC–MS

**Fig. 4:**
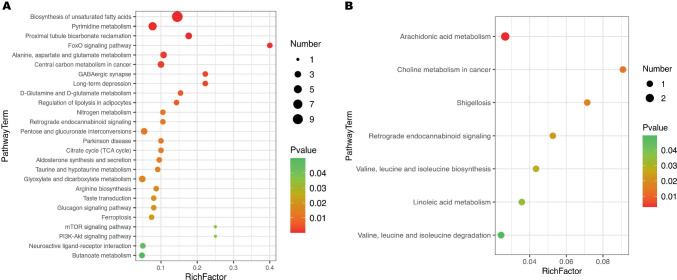
Bubble charts of KEGG pathways of DMs between mOSA and sOSA by GC–MS and LC–MS. Pathway analysis indicates biosynthesis of unsaturated fatty acids and FoxO signaling pathway are the most statistically enriched pathway from GC–MS (**A**). Arachidonic acid metabolism and choline metabolism in cancer are the most prominent pathways from LC–MS (**B**). Markedly, retrograde endocannabinoid signaling is the exclusive common pathway out of both GC–MS and LC–MS

In light of the aforementioned DMs, an enrichment analysis was conducted using the KEGG library. Figure 4A shows a total of 26 enriched pathways for DMs identified using GC–MS (p < 0.05). The top three pathways in sOSA were unsaturated fatty acid biosynthesis (p = 9.21*10E-11), pyrimidine metabolism (p = 0.00019), and proximal tubule bicarbonate reclamation (p = 0.00036). The FoxO signaling pathway (p = 0.0007) had the highest RichFactor (0.4), indicating that relevant DMs account for 40% of all metabolites of that. In addition, the PI3K-Akt signaling pathway, as the upstream of the FoxO pathway with a close impact on it, was also among the top 26 pathways.

Based on LC–MS, there were 7 enriched pathways of DMs, all presented in Fig. 4B (p < 0.05), including arachidonic acid metabolism, as a fundamental role with extensive effect in various diseases. Retrograde endocannabinoid signaling was a particularly intriguing finding in this study. As the single shared pathway, it could be retrieved from both LC–MS and GC–MS.

### ROS variations among various subgroups and their relationships with key DMs

**Fig. 5:**
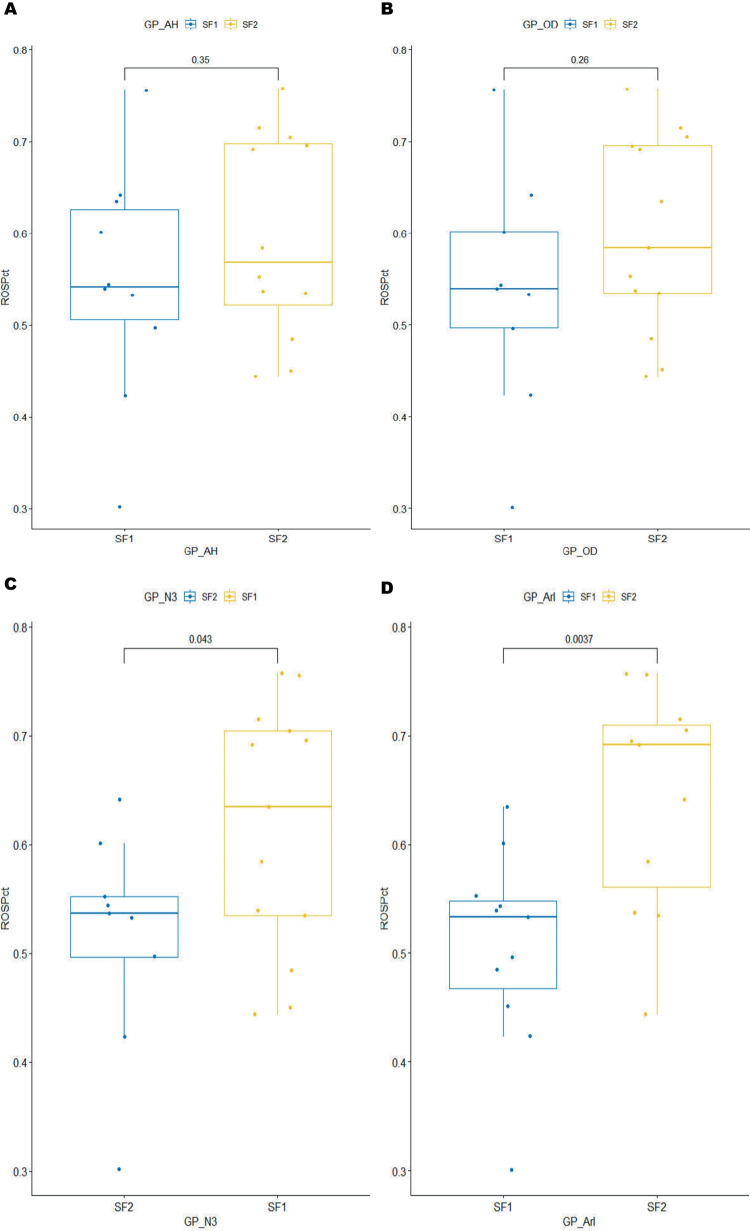
ROS Changes between OSA Subgroups according to Respiratory Events or Sleep Fragmentation. The comparation of ROS level in serum between mOSA and sOSA patients according to AHI (**A**) or ODI (**B**), without any significance shown. However, significant difference could be manifested between the subgroups (h-SF vs. l-SF) with high or low sleep fragmentation. In the subgroup with more N3 sleep duration, ROS level drops lower than in the subgroup with less N3 duration(p = 0.043, shown in (**C**). In the subgroup with higher arousal index(ArI > 30), ROS level elevates significantly than in the other(p = 0.0037, shown in (**D**)

Although ROS detection revealed no difference between subgroups based on AHI or ODI [Table 1, Fig. 5A, 5B], a significant difference was detected across subgroups with varying levels of sleep fragmentation (Figs. 5C, 5D). ROS concentration was lower in the subgroup with longer N3 duration (p = 0.043, cut-off value: 16 min, mainly according to 20% of a single sleep cycle at least), but increased significantly in the subgroup with a higher arousal index (p = 0.0037, cut-off value: 30/h, according to precedent research and grouping balance).

## Discussion

In this investigation, we demonstrated plasma metabolic profiling for young and middle-aged male patients with OSA using an integrated method of GC–MS and LC–MS untargeted metabolomics, as well as PSG and ROS detection. The findings revealed that 59 metabolites from GC–MS and 27 metabolites from LC–MS were significantly altered in sOSA patients when compared to mOSA. Extensive pathways were disrupted distinctly, with the only one in common being retrograde endocannabinoid signaling from both forms of metabolomics. Other enriched pathways included the FoxO signaling pathway and its upstream PI3K-Akt signaling pathway, different amino acid and nitrogen metabolism, lipid metabolism and TCA, particular synaptic function and neurodegeneration, renal tubular reabsorption, pyrimidine metabolism, and so on. Although there is no significant difference in ROS between mOSA and sOSA, the statistical significance of ROS between high and low fragmentation subgroups (N3 threshold: 16 min, arousal index: 30/h) was clearly established.

Various fatty acids and amino acids, glycosides or nucleosides, as well as small molecules of phosphates, intermediary metabolites in energy metabolism, and arachidonic acids, catecholamines, were revealed, as shown in previous OSA studies compared with the healthy controls [7–11], but fewer in sOSA patients comparing with the mOSA. In a mice model, H_2_S alleviates CIH-induced myocardial damage through PI3K/AKT/mTOR pathway, inhibiting oxidative stress and enhancing autophagy [13]. In another IH model in vitro and in vivo, GLP-1 analogue liraglutide’s protective mechanisms in combating cognitive deficits associated with CIH was via the Nrf2/HO-1 and MAPK/NF-κB pathways [14].The PI3K/Akt signaling system is essential for cell growth, apoptosis, translation, and metabolism. Activated Akt can directly phosphorylate a number of downstream effectors, inhibiting or activating their actions; one significant target is the FoxO family [15, 16]. The FoxO pathway regulates the cell cycle, apoptosis, oxidative stress resistance, and metabolism, and it is thought to play a significant role, primarily in response to stress circumstances rather than as a critical mediator of normal physiology [17]. An interesting finding in basic sleep research is that FOXO and the target of rapamycin (TOR) occupy opposing and agonistic positions within PI3K/Akt pathway. During the early stages of sleep, the insulin-like growth factor-1 (IGF-1) signaling pathway is activated. This activation occurs due to the secretory peaks of growth hormone (GH) and the downregulation of IGF-1 binding protein-1 (IGFBP-1), which in turn triggers the mitogen-activated protein kinase/extracellular signal-regulated kinase (MAPK/ERK) and PI3K/Akt signaling cascades. Phosphorylated Akt activates TOR, which promotes protein synthesis and growth processes, acting as a catalyst for growth and aging [18]. In the later stages of sleep, the IGF-1 signaling axis is downregulated. This process is mediated by declining GH levels, the somatostatin—and corticosteroid-induced upregulation of IGFBP-1. The absence of IGF-1 signaling inhibits PI3K/Akt activity, leading to the activation and nuclear translocation of FoxO. Once in the nucleus, FoxO regulates various cellular functions, including stress resistance and energy metabolism[T18]. Based on our study findings, the FoxO and PI3K/Akt pathways likely play a role in the pathophysiology of OSA.

The endocannabinoid system, which includes cannabinoid receptors, endogenous ligands, and enzymes that synthesize, degrade, and transport endocannabinoids, is found throughout the central and peripheral nervous systems, the endocrine system, the gastrointestinal tract, and inflammatory cells [19, 20]. It acts as a key modulator, regulating neurochemical systems, autonomic function, the circadian sleep–wake cycle, anxiety, and mood [19, 21, 22]. Preliminary research revealed its significance in OSA and insomina patients, as well as related models [21–23]. The best-studied endocannabinoids, 2-arachidonoyl-glycerol (2-AG) and anandamide, have a major impact on a variety of biochemical and physiological processes through cannabinoid receptors CB1R and CB2R. These mechanisms include thermoregulation, HPA axis activation, metabolism, memory consolidation, inflammation, and reward [21].

Previous research has found that endocannabinoids are increased in OSA patients compared to healthy persons [7]. The expression of enzymes involved in endocannabinoid production and breakdown, such as diacylglycerol lipase (DAGL) and monoacylglycerol lipase (MAGL), may change or enhance endogenous cannabinoid levels [24]. Furthermore, MAGL levels showed a moderate correlation with ArousaI, reflecting the severity of sleep fragmentation [25]. Wang and colleagues demonstrated that AEA and 2-AG levels increased in sOSA patients compared to the mild or moderate OSA category, with AEA linked with AHI and the homeostasis model of assessment for insulin resistance index (HOMA-IR) [26]. Furthermore, the principal conclusion of our investigation confirmed the participation of endocannabinoids in signaling pathways, while more widespread impacts on brain function could not be ruled out. Similarly, certain synaptic activities and neurodegenerative pathways, as shown in pathway enrichment by GC–MS, may have a latent influence on sleep-related neurologic disorders. A few studies concentrating on the administration of exogenous cannabinoids had ever demonstrated certain effects on sleep characteristics or AHI. However, due to the limitations in stability and reliability, AASM does not currently recommend cannabinoids as a treatment for OSA [21, 23].

Patients with OSA commonly undergo periods of intermittent hypoxia, similar to ischemia/reperfusion [27, 28]. This situation, characterized by repetitive cycles of oxygen deprivation and restoration, has a major impact on the development and progression of OSA and associated comorbidities, particularly cardiovascular illnesses and metabolic syndrome, owing to increased ROS production [27–31]. ROS, the most common result of oxidative stress, are transient intermediates in both normal and abnormal metabolism. And its production is a complicated and multifaceted process. ROS generation is influenced by a variety of parameters, including oxidative stress, mitochondrial function, nutritional and antioxidant status, age or life stage, and epigenetic changes [32]. Intrinsic changes to the electron transport chain (ETC), such as deficiencies in component assembly, mutations in ETC subunits, and ETC inhibition, can also increase ROS production [33]. Differences in redox disruption appeared to be related to both aging and physiologic exercise [34]. Mitochondrial uncoupling of oxidative phosphorylation, or proton leak, reduces the electrochemical proton gradient (Δp) and accounts for up to 25% of basal metabolism in the presence of oxidative stress, such as diabetes, drug resistance in tumor cells, ischemia–reperfusion injury, or aging. Variations in mitochondrial uncoupling proteins (UCP) expression may influence the effectiveness of ROS generation in the cardiovascular system [35]. Notably, OSA may drive ROS production through multiple distinct mechanisms beyond classical oxidative stress pathways. First, recurrent oxygen desaturation events induce sleep fragmentation and chronic sleep deprivation, which disrupt redox homeostasis via circadian rhythm dysregulation. Second, OSA-associated systemic inflammation promotes ROS generation through proinflammatory cytokine-mediated activation of NADPH oxidases (NOX), particularly in vascular endothelium. Furthermore, metabolic perturbations secondary to IH—including altered mitochondrial respiration and sleep deprivation-induced lipolysis—create a pro-oxidant microenvironment that perpetuates ROS overproduction [36]. Our findings show that sleep fragmentation can cause noticeable changes in ROS levels between subgroups of patients, regardless of AHI or ODI. Young and middle-aged flies have been shown to have preserved neuroprotective mechanisms in response to sleep fragmentation. In cases of poor sleep quality, elevated neuronal ROS levels may promote neuronal insulin signaling by blocking cytochrome C release, a mechanism that protects neurons from death caused by unfolded protein responses (UPR) [37]. This finding shows that, even in the absence of severe AHI or ODI, sleep fragmentation may play an unignorable role in ROS formation.

In our study sOSA group showed a significantly higher BMI compared to the mOSA, which displayed the robust link between obesity and OSA. Epidemiologically, 70% of OSA patients are obese, and higher BMI correlates with more severe OSA, particularly in males and younger populations [3, 36], consistent with our study. Cervical fat accumulation narrows the oropharynx and diminishes upper airway traction, while obese men with OSA exhibit less weight loss after a one-year dietary and exercise intervention compared to similarly obese men without OSA. Emerging evidence highlights intermediate mechanisms—including oxidative stress, endothelial dysfunction, insulin resistance, and inflammation—as key contributors to the concurrence of both [3, 36]. In a longitudinal cohort study of 544 participants, OSA was identified as an independent risk factor for developing obesity-associated T2DM, with a significant positive correlation between AHI and BMI. This association remained statistically significant after adjusting for confounding variables (age, sex, BMI, race, etc.) [36]. Notably, comorbidities such as heart failure, stroke, and COPD could not be fully attributed to BMI or obesity [3], which further highlights OSA's role as an independent risk factor for systemic disorders. However, the bidirectional and synergistic interplay between obesity and OSA renders the efforts to differentiate their independent impacts on complications, particularly in cohort with limited sample sizes. We must acknowledge our current study's limitations. The sample size is small, and all of the cases are male and of Chinese Han ethnicity. The possibility of sample bias and individual differences may have an impact on its generalizability.

## Conclusion

Our preliminary findings suggest that the retrograde endocannabinoid signaling pathway, which is shared by both GC–MS and LC–MS, may play a role in sleep disorders and other systemic consequences, including brain function, though more research is needed to elucidate its exact effect and mechanism. The comprehensive metabolomics investigation using GC/LC–MS revealed considerable metabolic changes, with important pathways implicated, such as the FoxO signaling pathway and its proximal PI3K-Akt signaling network, among others. Sleep fragmentation, in addition to AHI and ODI, may be a useful predictor of the degree of oxidative stress in young and middle-aged OSA patients.

## Data Availability

The data are available from the corresponding author on reasonable request.
